# The Role of Nuclear Factor Kappa B (NF-κB) in Development and Treatment of COVID-19: Review

**DOI:** 10.3390/ijms23095283

**Published:** 2022-05-09

**Authors:** Monika Gudowska-Sawczuk, Barbara Mroczko

**Affiliations:** 1 Department of Biochemical Diagnostics, Medical University of Bialystok, 15-269 Bialystok, Poland; mroczko@umb.edu.pl; 2 Department of Neurodegeneration Diagnostics, Medical University of Bialystok, 15-269 Bialystok, Poland

**Keywords:** SARS-CoV-2, COVID-19, inflammation, Nuclear Factor kappa B (NF-κB), inflammatory factors

## Abstract

Severe acute respiratory syndrome coronavirus 2 (SARS-CoV-2) causes Coronavirus Disease 19 (COVID-19), a disease that has affected more than 500 million people worldwide since the end of 2019. Due to its high complications and death rates, there is still a need to find the best therapy for SARS-CoV-2 infection. The dysregulation of the inflammatory response in COVID-19 plays a very important role in disease progression. It has been observed that abnormal activity of Nuclear Factor kappa B (NF-κB) is directly associated with, inter alia, increased synthesis of proinflammatory factors. Therefore, this review paper focuses on the functions of NF-κB in the development of SARS-CoV-2 infection and potential application of NF-κB inhibitors in COVID-19 immunotherapy. A comprehensive literature search was performed using the MEDLINE/PubMed database. In the current review, it is highlighted that NF-κB plays important functions in the modulation of an adaptive inflammatory response, including inducing the expression of proinflammatory genes. Increased activation of NF-κB in SARS-CoV-2 infection was observed. The association between NF-κB activation and the expression of SARS-CoV-2 structural and non-structural proteins were also reported. It was observed that modulation of NF-κB using, e.g., traditional Chinese medicine or glucocorticosteroids resulted in decreased synthesis of proinflammatory factors caused by SARS-CoV-2 infection. This review summarizes the role of NF-κB in COVID-19 and describes its potential immunotherapeutic target in treatment of SARS-CoV-2 infection. However, indisputably more studies involving patients with a severe course of COVID-19 are sorely needed.

## 1. Introduction

**Figure 1:**
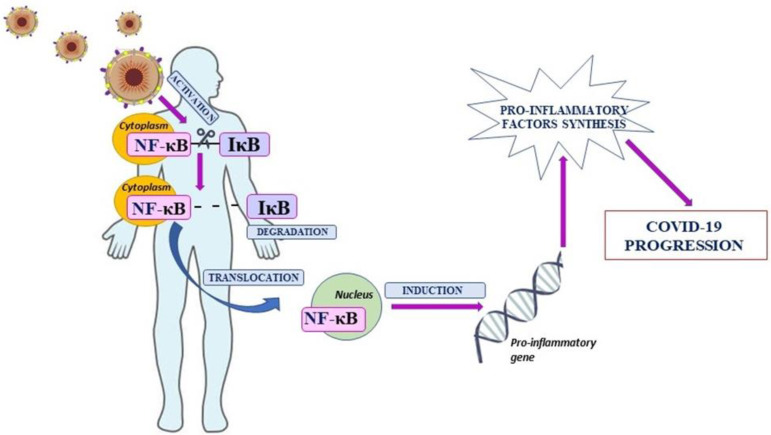
The role of Nuclear Factor kappa B in SARS-CoV-2 infection.

Coronavirus Disease 19 (COVID-19) is a disease caused by the coronavirus SARS-CoV-2, originating in Wuhan, China. The COVID-19 name was given by the World Health Organization where the number 19 indicates the year of the appearance of the virus—2019 [1,2]. The SARS-CoV-2 virus belongs to the family of coronaviruses that are mainly characteristic for animals. However, coronaviruses have an ability to mutate and, as a result, can infect other species including humans. The infection manifests in many forms—from asymptomatic to, e.g., severe pneumonia and respiratory failure, or even multiple organ failure and death [3,4]. The response of the immune system to the SARS-CoV-2 infection does not differ significantly from that observed in infections caused by other viruses. Viral antigens are identified as foreign elements through non-specific immunity, which is the primary line of defense against the pathogen [5]. In addition, as a result, the production of proinflammatory factors, including cytokines, starts. Those mediators have an impact on the proliferation and stimulation of cells involved in the immune response. The overproduction of cytokines and the cytokine storm lead to dysregulation of the immune system, intensification of inflammation, and homeostatic imbalance [6,7,8]. It was observed that impaired modulation of genes involved in processes directly associated with inflammation, including genes encoding cytokines and chemokines, is dependent on the abnormal activation of Nuclear Factor kappa B (NF-κB) [9]. NF-κB was discovered in B cells by Sen and Baltimore in 1986. It was found in mouse lymphocytes as a critical mediator involved in the transcription of the murine immunoglobulins’ light chains [10,11]. The mammalian NF-κB family consists of five transcription factors which are divided into two groups. The first is a complex of three mature and active proteins: c-Rel, RelA (p65), and RelB, and the second consists of inactive precursor proteins: NF-κB1 (p50/p105) and NF-κB2 (p52/p100). It was revealed that NF-κB proteins form dimeric structures among which only heterodimers have a biological effect. Except for RelB, which forms heterodimers, other proteins also form homodimers. The interaction of NF-κB dimers with specific inhibitors (IκB) is the reason for retention in the cytoplasm, but three specific signaling pathways lead to induction of NF-κB. It stimulates the nuclear translocation and activation of genes involved in inflammation that are under control of NF-κB [12,13]. Going forward, it was revealed that increased activation of NF-κB leads to markedly elevated production of proinflammatory mediators and finally the cytokine storm [14]. Moreover, NF-κB is an important regulator of T cells and other innate immune cells’ differentiation and activation. Therefore, dysregulation of NF-κB can lead to an uncontrolled and pathogenic inflammatory response [12,15]. Interestingly, it was observed that upregulation of NF-κB is involved in the development of SARS-CoV-2 infection (Figure 1) [15,16,17]. The SARS-CoV-2 infection may induce an uncontrolled inflammatory response, and for this reason it is necessary to find compounds able to inhibit inflammation caused by, inter alia, NF-κB signaling pathway activation. Therefore, the aim of this review was to discuss the role of nuclear factor kappa B in the pathogenesis and treatment of COVID-19.

## 2. Material and Methods

**Figure 2:**
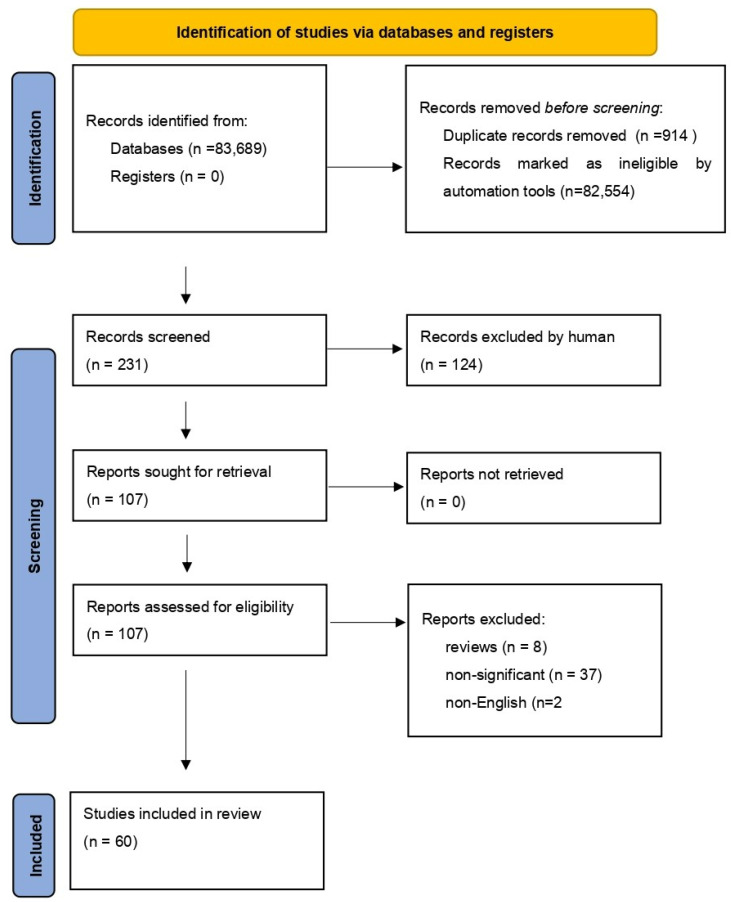
The PRISMA Flow Diagram.

We performed a comprehensive literature search covering the period up to the 15 April 2022. We used the MEDLINE/PubMed database with the following search strategy: key word “NF-κB” (83,689 studies). Then, we used the key words “NF-κB AND COVID-19”, and a total of 231 papers were found. In the next step, we limited studies to studies in English, and we excluded duplicates or all non-significant papers (i.e., papers that did not concern NF-κB or COVID-19). Finally, 60 publications were included in the review. All steps are presented in the PRISMA Flow Diagram (Figure 2) [18].

## 3. Results and Discussion

### 3.1. The Association between SARS-CoV-2 Proteins and NF-κB Signaling Pathway

#### 3.1.1. Spike Protein

The accurate mechanism of the SARS-CoV-2-induced NF-κB pathway is still poorly understood. It was observed that the spike receptor-binding domain (S-RBD) and integrin α5β1 may induce nuclear translocation of NF-κB through IκBα degradation. Knowing that inflammatory genes depend on NF-κB, Robles et al. tried to investigate whether binding of the spike (S) protein to α5β1 in vascular endothelial cells (EC) is essential to activation of the inflammatory response. The activation of the NF-κB signaling pathway accompanied by α5β1 was associated with leukocyte adhesion to ECs. Moreover, the increased expression of coagulating factors (tissue factor, factor VIII) or proinflammatory cytokines and increased permeability of EC monolayer were observed. A5β1-induced NF-κB was also associated with increased expression of angiotensin-converting enzyme 2 (ACE2). Importantly, expression of ACE2 upregulated by Spike was blocked by anti-α5β1 antibodies and inhibitors of NF-κB1. Therefore, the modulation of NF-κB may have a protective effect on EC viral infection by a reduction in coronavirus receptors, mainly ACE2 [19]. The induction of NF-κB and, hence, increased synthesis of proinflammatory cytokines were described by Neufeldt et al., also. Total RNA from infected lung ECs was analyzed using microarray. Gene analysis revealed changes and upregulation of NF-κB and IL-6 STAT3 genes. Transcriptional activation of the NF-κB pathway resulting in cytokine production indicated that EC infection may contribute to the cytokine storm observed in a severe course of the SARS-CoV-2 infection. The activation of NF-κB was followed by cyclic GMP–AMP synthase (cGAS)—stimulator of interferon genes (STING) pathway—and it was associated with reduced activation of interferon regulatory transcription factor 3 and the IFN axis [20]. Initially, STING was considered as a factor involved in the immune response against tumors [21]. However, it has also an ability to control infections caused by viruses, and it plays a crucial role in lung inflammation [22]. Therefore, NF-κB activation facilitated by a STING may potentiate the inflammatory response against SARS-CoV-2. Unfortunately, the imbalanced inflammatory response and cytokine storm in lungs’ ECs were found to intensify the symptoms greatly [20,22].

An interesting study performed by Khan et al. demonstrated the effect of S SARS-CoV-2 protein on toll-like receptor 2 (TLR-2)-induced NF-κB. It was observed that extracellular Spike protein induces production of inflammatory factors including chemokines or TNF-α in human and mouse lung cells. On the other hand, no inflammatory response was observed in response to intracellular Spike protein. Interestingly, this study showed that mice with macrophages devoid of TLR-2 had downregulated activity of NF-κB and thus decreased synthesis of IL-1β, IL-6, and TNF-α. Moreover, it should be pointed that the activation of NF-κB requires the creation of TLR-2 heterodimers with TLR-6 or TLR-1. So, modulation of TLR-2, the immune sensor for S protein, as a novel therapeutic target in COVID-19 should be considered [23].

#### 3.1.2. Nucleocapsid Protein

It was reported that the process of, e.g., acute lung injury (ALI) is also dependent on the expression of nucleocapsid (N) protein, but the mechanism is not fully understood. The elevation of SARS-CoV-2 N protein is observed even in early stages of infection. On the study on mice and bone marrow-derived macrophages (BMDMs) exposed to N protein, it was observed that N protein may induce lung injury, and the effect is dose-dependent. Moreover, its overexpression parallels to upregulation of NF-κB phosphorylation, and the induction of NF-κB was observed at 10 min after exposure. At the same time, it was observed that in vitro NF-κB activation promotes polarization of M1 macrophages and that N protein increases the mRNA expression of proinflammatory cytokines. On the other hand, N protein and NF-κB phosphorylation are regulated by antibodies against the SARS-CoV-2 nucleocapsid while it is inhibited by anti-nucleocapsid antibodies or denaturation of N proteins. More specifically, SARS-CoV-2 N protein caused phosphorylation of NF-κB in the lungs whereas its inhibitor efficiently alleviates the impact of SARS-CoV-2 on ALI [24].

#### 3.1.3. Non-Structural Proteins

The activation of NF-κB via previously mentioned α5β1 and cGAS-STING does not exclude other induction factors. Makiyama et al. evaluated whether nucleoporin 62 (NUP62) has an association with mechanisms of the host’s immune response. It was observed that NUP62, a structural component of nuclear pore complexes, has an ability to interact with non-structural SARS-CoV-2 protein 9 (NSP9). Moreover, it was revealed that increased NSP9 expression correlates with inhibition of NUP62. Going forward, NUP62 depletion and overexpression of NSP9 may lead to impaired nuclear translocation of NF-κB [25].

The literature revealed also the participation of NSP5 in activation of the NF-κB signaling pathway. Findings from Li et al.’s study focused mainly on the role of NSP5 on inflammation caused by SARS-CoV-2 infection. The scientists reported that NSP5 can induce the synthesis of proinflammatory cytokines including IL-2 in Calu-3 and THP1 cells. Furthermore, evidence supporting the activation of NF-κB by NSP5 was also revealed. Interestingly, the induction of NF-κB was preceded by SUMOylation which has been recognized as a regulator of biological processes including transcription and protein subcellular localization. Moreover, Li et al. demonstrated that SUMOylation of the mitochondrial antiviral-signaling protein (MAVS) increases its stability and level. On the other hand, inhibition of SUMOylation can reduce activation of NF-κB through NSP5 and, hence, the cytokine storm and inflammation [26].

#### 3.1.4. ORF Proteins

It was observed also that ORF conserved proteins of SARS-CoV-2 are associated with virulence and coronavirus infectivity. In addition, ORF3a, ORF7a, membrane (M), and nucleocapsid subunits have an ability to induce the NF-κB pathway by at least two-fold. However, only ORF7a induces the NF-κB dependent synthesis of INF-γ, cytokines (e.g., IL-6, IL-8, IL-10, IL-1, TNF-α), and chemokines (e.g., CXCL9, CCL11, CCL20, CCL21). The NF-κB activation was also SARS-CoV-2 dose dependent, as evidenced by a positive correlation between amounts of viral proteins. Moreover, cytokines’ concentrations were markedly elevated in patients with a severe course of COVID-19. Therefore, it was suggested that ORF7a modulation may be a potential therapeutic target involved in activation of NF-κB and, as a result, the inflammatory response in SARS-CoV-2 infection [27].

#### 3.1.5. Treatment

It is well known that NF-κB is involved in inflammation, and the increased inflammatory response dependent on the SARS-CoV-2 dose was observed. Abnormal activity of NF-κB is accompanied by the increased release of proinflammatory factors, including cytokines. The cytokine storm can lead to development of inflammation and COVID-19 progression. Contrarily, reduced activity of NF-κB may inhibit cytokine release and development of SARS-CoV-2 infection. Therefore, it was suggested that anti-SARS-CoV-2 therapy should focus on inflammatory response extinction through, inter alia, modulation of NF-κB [28,29,30,31,32,33].

#### 3.1.6. Natural Pharmaceuticals

ACE2 is one of SARS-CoV-2 receptors which is expressed in various organs, e.g., the lungs, liver, central nervous system (CNS), or vascular system. Well, actually, that is why SARS-CoV-2 can spread and attach almost to the whole human body [34,35,36]. It was observed that an interaction between SARS-CoV-2 spike protein and ACE2 results in NF-κB activation. The binding of the S Receptor Binding Domain (S-RBD) to ACE2 results in the cytokine storm through NF-κB or mitogen-activated protein kinase (MAPK).

Sharma et al. evaluated the effect of curcumin-encapsuled polysaccharide nanoparticles (CUR-PS-NPs) on coronavirus induced synthesis of proinflammatory factors. Using the ELISA method, they observed that CUR-PS-NPs inhibit the interaction between ACE2 and SARS-CoV-2. Moreover, it was observed that infected liver (Huh7.5) and lung (A459) epithelial cells exhibit increased levels of phosphorylated p38 MAPK and NF-κB, and decreased levels of its major inhibitor protein—IκBα. On the other hand, the use of CUR resulted in inhibition of SARS-CoV2 mediated activation of the MAPK/NF-κB axis. In addition, going further, production of the most important proinflammatory cytokines, IL-6 and IL-8, was reduced. Summarizing, the study revealed that Nanocurcumin effectively inhibits the cytokine storm triggered by SARS-CoV-2 and activation of the NF-κB signaling pathway [37].

The management options for S1-induced inflammation are limited and still controversial. The objective of Olajide et al.’s study was to investigate the outcome of *Garcinia kola* and garcinoic acid treatment. *Garcinia kola* is a West African plant with a wide range of functions including regulation of anti-inflammatory, antioxidant, and antithrombotic activity [38,39]. Moreover, garcinoic acid isolated from *Garcinia kola* is an analogue of vitamin E with the ability to inhibit the inflammatory response. It was found that both *Garcinia kola* and garcinoic acid reduced overexpression of p65 NF-κB and p65 IκBα, and also the NF-κB DNA binding capacity caused by Spike 1 protein of SARS-CoV-2. Blocking the NF-κB signaling by the drugs inhibits the secretion of the most proinflammatory cytokines: IL-6, IL-8, IL-1β, and TNF-α in PBMCs. Therefore, lung epithelial cells’ damage induced by the increased inflammatory response and NF-κB activity may be reduced by the natural pharmaceuticals described above [38].

COVID-19 progression and acute respiratory distress syndrome (ARDS) have an association with increased levels of cytokines, including also tumor necrosis factor (TNF). TNF is a proinflammatory and pleiotropic cytokine produced by many cells during inflammation [40,41,42,43]. TNF has been implicated in bronchial overactivity, pulmonary fibrosis, and respiratory system damage. It was observed that chronic and pathologic inflammation is associated with dysregulation of the TNF-induced NF-κB signaling transduction pathway. Therefore, NF-κB inhibitors may have an effect on TNF neutralization. Both TNF and NF-κB blockers may probably lead to a reduction in the cytokine storm [44]. Rehan et al. evaluated the influence of *Andrographis paniculate* on the inhibition of the TNF-induced NF-κB1 signaling pathway. Using the “host response signature network”, which consists of 36 genes, it was revealed that TNF-induced NF-κB determines to a large extent the cytokine storm. They observed that TNF is associated with NF-κB and some proinflammatory mediators including IL-6, CCL20, MMP-9, or CXCL1. Molecular docking analysis performed by authors revealed also that Andrographolide may bind to crucial proteins and hence block what is responsible for the cytokine storm, TNF, and NF-κB1 [41].

It is well known that the Mediterranean diet is rich in polyphenols that are found in, e.g., fruits, vegetables, herbs, or cereals. It was speculated that the Mediterranean diet and so polyphenols may regulate the inflammatory response. It was observed that Naringenin (NAR), which belongs to polyphenols and which is found in herbs and fruits, may be effective in the management and prevention of diabetes, metabolic syndrome, or liver damage. Moreover, knowing that NAR has immunomodulatory properties, it was suggested that it has a potential therapeutic effect in COVID-19. The immunomodulatory and antiviral effect of this polyphenol is associated with the regulation of, inter alia, the NF-kB signaling pathway described in this review. It was observed that NAR reduces leukocyte recruitment through the inhibition of NF-κB. Going forward, naringenin reduced the synthesis of cytokines (TNF-α, IL-1β, IL-6, IL-12) that resulted in a decreased immune response. Taken together, these findings suggest that NAR is related to decreased activation of NF-κB, production of proinflammatory factors, and, hence, reduced inflammation caused by, e.g., SARS-CoV-2 infection [45,46,47].

#### 3.1.7. Heat Shock Protein 90

It is well known that subunit 1 of Spike protein (S1SP) may induce lung endothelial activation and damage. Moreover, it was found that heat shock protein 90 (HSP90) is involved in many cellular processes including modulation of the NF-κB or STAT3 signaling pathway leading to inflammation and cells’ dysfunction. Therefore, Colunga Biancatelli et al. tried to evaluate the effect of HSP90 inhibitors on endothelial barrier dysfunction through regulation of, e.g., NF-κB inhibitor—IkBα. The authors observed that HSP90 inhibitors (AT13387, AUY-922) prevented S1SP-induced increased permeability and dysfunction of the endothelial barrier. This therapeutical effect was associated with the modulation of NF-κB and that HSP90 inhibitors reduced IkBα activation. Therefore, it was speculated that HSP90 may have potential therapeutical significance, and the treatment with HSP90 inhibitors might be a promising strategy in the acute lung injury and endothelial dysfunction caused by SARS-CoV-2 infection [48].

#### 3.1.8. Vitamin D

Unfortunately, it was observed that thrombosis is a frequent complication of COVID-19. The evidence that vitamin D may prevent thrombosis in SARS-CoV-2 infection was supported by Cimmino et al. [49]. As is known, vitamin D plays a crucial role in the regulation of the immune response, and it is involved in growth factors and cytokines’ synthesis [49,50]. Taking into account that IL-6 is both a pro- and anti-inflammatory cytokine, the impact of vitamin D on adhesion molecules (CAMs) and the tissue factor (TF) in the endothelial cells stimulated by IL-6 was evaluated. It was observed that vitamin D reduced TF procoagulant activity in patients treated with IL-6. In addition, as can be expected, vitamin D prevented the proinflammatory and prothrombotic effect by regulation of NF-κB. What is worth pointing out is that the NF-κB signaling pathway may be involved in the thrombosis, because of binding sites for NF-κB that are presented in the activator of the extrinsic coagulation pathway—the promotor of the TF. Interestingly, translocation of NF-κB to the nucleus was significantly inhibited in the human umbilical vein endothelial cells preincubated with vitamin D and before stimulation with IL-6 [49,50,51].

#### 3.1.9. Traditional Chinese Medicine

Traditional Chinese Medicine (TCM) is another option to treat COVID-19. It was suggested that one of the TCMs, Qingwenzhike (QWZK), may be an effective therapy in ALI caused by infection, e.g., SARS-CoV-2. The effectiveness of QWZK was evaluated on a rat model of infection induced by lipopolysaccharides (LPS). It is well known that LPS can bind to toll-like receptors. TLRs are activated to support an innate immune response. The action of, inter alia, TLR 3 or TLR4 leads to activation of NF-κB and the release of proinflammatory mediators [52,53,54]. In the study conducted by Zhang et al., it was observed that QWZK has a protective effect on the overexpression of TLR4 and also NF-κB. The inhibition of TLR4 and NF-κB resulted in reduced production of TNF-α, IFN-γ, IL-6, IL-18, IL-1β, and monocyte chemoattractant protein (MCP-1). Moreover, the expression of NF-κB was significantly lower in the rat group treated with QWZK in comparison to dexamethasone or the recovered group. A similar correlation was observed with QWZK and downregulation of proinflammatory cytokines. So, the results presented by authors indicate that QWZK is a promising therapeutic option for NF-κB modulation and, therefore, ALI caused by, e.g., SARS-CoV-2 [53].

The effect of the Liu Shen capsule (LS) on SARS-CoV-2 was recently assessed. In particular, it is known that LS has an anti-inflammatory and antiviral activity. In fact, the use of the Liu Shen capsule has been proven to be effective in regulating the host’s immune response. Ma Q et al. measured the effect of the LS on those infected by SARS-CoV-2 human hepatocellular carcinoma cell lines (Huh-7) and African green monkey kidney epithelial cells (Vero E6). The reduction in the coronavirus-induced cytopathic effect was observed for treatment with the LS. In addition, the proof of this was a drop in the mRNA levels of proinflammatory mediators such as IL-1β, IL-6, CCL2, CXCL10, and TNF-α. Importantly, the use of the LS was associated with inhibition of p-NF-κB p65 and elevated expression of IκBα. Thus, the LS seems to possess anti-SARS-CoV-2 efficacy through the NF-κB signaling pathway [55].

The potential effectiveness of another TCM against coronaviruses was evaluated. Yindan Jiedu (YDJDG), Cangma Huadu (CMHD), and Jinzhen granules (JZ) are among TCMs whose efficiency in COVID-19 treatment was investigated. The CMHD effect was evaluated on pneumonia induced by Human coronavirus 229E (HCoV-229E), YDJDG against SARS-CoV-2, whereas the JZ effect was tested on two coronaviruses. Regardless of the type of coronavirus, studies revealed decreased induction of proinflammatory cytokines including the above mentioned IL-1β, IL-6, and TNF-α by the drugs. This can be explained by the fact that TCMs may have an effect on NF-κB on which this review focuses. It was stated that CMHD, YDJDG, and JZ have an ability to inhibit the expression of p-NF-κB p65 in lungs, and, therefore, lung damage may be suppressed. Moreover, the study on YDJDG performed a detailed analysis which revealed that 25 of those analyzed and associated with the NF-κB pathway or IκB1 genes were markedly enriched. Based on these data, it was suggested that both tested granules have antiviral activity and the ability to inhibit the inflammatory response caused by coronaviruses through NF-κB signaling pathway modulation [56,57,58].

#### 3.1.10. Glucocorticosteroids

It is well known that the NF-κB p65 and NF-κB p50 heterodimer plays a crucial role in the inflammatory response against SARS-CoV-2, and it was revealed that the glucocorticoid receptor (GR) is its physiological antagonist involved in anti-inflammatory processes. Therefore, Spinelli et al. evaluated in the PBMCs’ expression level of genes: p65 and its new spliced variant which contains a previously unknown exon located before the exon 0 of p65 (p65 iso5). It was observed that the expression of both was significantly higher in patients with SARS-CoV-2 infection in comparison to healthy controls and that p65 is able to activate transcription trough NF-kB elements ten times more than p65 iso5. Surprisingly, the new isoform of p65 has an ability to enhance the GR anti-inflammatory response. In addition, p65 iso5 binds dexamethasone, and therefore it doubly enhances the therapeutic effect mediated by glucocorticosteroids. Taking into account the above, it was speculated that a new p65 isoform could modulate anti-inflammatory processes in COVID-19. On the other hand, the heterodimer of p65 or p50 with p65 iso5 induces the transcriptional activation of proinflammatory (IL-6 and TNF-α) genes more effectively than the classical NF-kB heterodimers. So, unfortunately p65 iso5 may be also involved in the cytokine storm and COVID-19 progression [59].

#### 3.1.11. Vasoactive Intestinal Peptide

In addition, from the therapeutic point of view, vasoactive intestinal peptide (VIP) seems to be promising. The neuropeptide VIP plays an important role in homeostasis of the immune system, and it has mainly anti-inflammatory activity [60,61]. It was observed that in severe COVID-19, VIP levels are markedly elevated. Moreover, its concentration negatively correlates with mortality of patients with SARS-CoV-2 infection. Importantly, VIP with pituitary adenylate cyclase activating peptide (PACAP) reduced the SARC-CoV-2 RNA expression and NF-kB activation in monocytes. There was also an association between VIP and inhibition of viral replication in lung epithelial cells. Furthermore, VIP was involved in activation of cAMP-response element binding protein (CREB) which is a negative regulator of NF-kB. These data suggest that VIP decreases activation of NF-kB and its target genes, including cytokines. Hence, it is speculated that inhibition of NF-kB by VIP is a promising therapeutic target for patients with SARS-CoV-2 infection [60].

#### 3.1.12. N-(1-carbamoyl-2-phenyl-ethyl) Butyramide

The effect of N-(1-carbamoyl-2-phenyl-ethyl) butyramide (FBA) on SARS-CoV-2-induced inflammation in the human small intestine, as well as enterocytes, was evaluated. It was observed that FBA has an ability to modulate genes associated with the anti-SARS-CoV-2 response. Firstly, it was revealed that FBA significantly reduced coronavirus entry into the host cell through ACE2, neuropilin 1 (NRP-1), and transmembrane serine protease 2 (TMPRSS2). Secondly, FBA blocked NF-kB overexpression, and, therefore, the cytokine storm was inhibited [62]. On the other hand, nuclear factor 2 (Nrf2) is a very important factor responsible for the anti-inflammatory response [63]. It was observed that nuclear factor 2 (Nfr2) that can be modulated through the NF-kB pathway was increased due to FBA. Moreover, in parallel to NF-kB, FBA reduced the expression of monocyte chemoattractant protein-1 (MCP-1), IL-15, and TNF-α in human enterocytes [64]. Similar to FBA, it was observed that diosmectite inhibits the interaction between ACE-receptors on enterocytes and Spike protein of SARS-CoV-2. In addition, diosmectite reduced inflammation and NF-kB activation. As a result of NF-kB inhibition, the synthesis of proinflammatory CXCL10 was observed. Knowing that chemokine CXCL10, through CXCR3, may be involved in the pathogenesis of COVID-19, it is important to find the best and most effective inhibitor of NF-kB [62].

A summary of proposed tested therapeutical doses of selected drugs described in this review are presented in Table 1.

## 4. Conclusions

Development and progression of SARS-CoV-2 infection is caused by dysregulation of the immune response and, e.g., overproduction of cytokines. It was revealed that intensification of inflammation is associated with impaired modulation of genes encoding proinflammatory factors by NF-kB. Therefore, during the last 2 years, the NF-kB signaling pathway was extensively investigated as a very important factor in the immune response in COVID-19. The overactivity of NF-kB leads to a systemic and long-term inflammatory response, increasing disease severity. This review focused on the role of NF-kB in the development and treatment of SARS-CoV-2 infection. Analyzed studies revealed that COVID-19 is associated with an increased activation of NF-kB. NF-kB activity depends also on SARS-CoV-2 structural and non-structural proteins. Moreover, the expression of SARS-CoV-2 proteins correlated with upregulation of NF-kB. In addition, overactivity of NF-kB was the reason for increased synthesis of cytokines and chemokines, and so the disease progression. It was observed that use of NF-kB inhibitors, including some natural pharmaceuticals, traditional Chinese medicine, or glucocorticosteroids, resulted in a reduction in the SARS-CoV-2-mediated NF-kB pathway. Going, further, production of proinflammatory factors, e.g., IL-6, IL-1β, or TNF-α, was reduced. These findings suggest that the abnormal inflammatory response in COVID-19 could be modulate by NF-kB, but more studies involving patients with a severe course of SARS-CoV-2 infection are sorely needed. Summarizing, NF-kB plays a very important role in the progression of SARS-CoV-2 infection and its transformation into severe COVID-19. Finally, modulation of NF-kB by specific inhibitors seems to be a promising target for COVID-19 immunotherapy.

## Figures and Tables

**Table 1:** Proposed therapeutical doses of selected drugs.

Drug	Tested Doses	References
Nanocurcumin	0.001, 0.01, 0.1, 0.2, 0.5, 5.0 μM	[37]
*Garcinia kola*	6.25, 12.5, 25 μg/mL	[38]
Garcinoic acid	1.25, 2.5 and 5 μM	[38]
Naringenin	50.0, 150.0 mg/kg	[46]
AUY-922, AT13387	2.0 μM	[48]
Qingwenzhike	3.0, 6.0, and 12.0 g/kg/day; 71.5 g/day	[53]
Liu Shen capsule	0.50, 1.00, 2.00 μg/mL	[55]
Yindan Jiedu granules	12.0 and 24.0 g/day	[56]
Cangma Huadu granules	12.1, 6.05 and 3.03 g/kg/day	[57]
Jinzhen granule	224, 448, 896 mg/kg/day	[58]
Diosmectite	100 mg/mL	[62]

## Data Availability

Not applicable.
